# Supplementary material for: Mechanisms governing the pioneering and redistribution capabilities of the non-classical pioneer PU.1
Source: Nat Commun. 2020 Jan 21;11:402. doi: 10.1038/s41467-019-13960-2 (PMC6972792; doi:10.1038/s41467-019-13960-2)
Supplement: Supplementary file 7 — Source data [file 41467_2019_13960_MOESM7_ESM.zip › Source_Data/FigureS2/FigureS2B_MotifScanOutput/KmeansCluster5/homerResults/motif14.similar.html]

motif14

## Information for motif14

T
G
C
A
T
G
C
A
A
G
C
T
T
A
G
C
C
T
G
A
C
G
A
T
C
T
A
G
C
A
T
G
G
T
A
C
C
T
A
G
A
T
C
G
G
T
C
A
C
G
T
A
  
Reverse Opposite:  

C
G
A
T
C
A
G
T
A
T
G
C
G
A
T
C
C
A
T
G
G
T
A
C
A
G
T
C
G
C
T
A
G
A
C
T
A
T
C
G
T
C
G
A
A
C
G
T
A
C
G
T
  

|  |  |
| --- | --- |
| p-value: | 1e-66 |
| log p-value: | -1.531e+02 |
| Information Content per bp: | 1.667 |
| Number of Target Sequences with motif | 185.0 |
| Percentage of Target Sequences with motif | 4.65% |
| Number of Background Sequences with motif | 335.9 |
| Percentage of Background Sequences with motif | 0.78% |
| Average Position of motif in Targets | 96.7 +/- 67.4bp |
| Average Position of motif in Background | 102.8 +/- 67.6bp |
| Strand Bias (log2 ratio + to - strand density) | 0.2 |
| Multiplicity (# of sites on avg that occur together) | 1.01 |
| Motif File: | file (matrix) reverse opposite |

### Similar de novo motifs found

|  |  |  |  |  |  |  |  |
| --- | --- | --- | --- | --- | --- | --- | --- |
| Rank | Match Score | Redundant Motif | P-value | log P-value | % of Targets | % of Background | Motif file |
| 1 | 0.780 | T C G A C G A T C T A G C T A G A G T C C T A G C T A G T G C A C T G A C T A G C T A G | 1e-57 | -132.732769 | 5.43% | 1.28% | motif file (matrix) |
| 2 | 0.767 | C G T A T A C G A G T C C G T A C G A T A T C G C T A G C T A G A C T G C T A G C G T A G T C A | 1e-47 | -109.738617 | 2.34% | 0.23% | motif file (matrix) |
| 3 | 0.786 | C A G T T A G C G T A C A T C G A G T C T A G C C G T A A G C T C T A G | 1e-44 | -102.928654 | 11.43% | 5.34% | motif file (matrix) |
| 4 | 0.764 | T C A G T G A C A G T C A C G T A G C T A G T C A G C T C T A G A T G C A G T C C G T A A C G T | 1e-37 | -86.628894 | 2.39% | 0.36% | motif file (matrix) |
| 5 | 0.788 | A T G C A G T C T A G C G T A C A G T C C T G A A G C T C T A G T C G A A G C T A G C T | 1e-36 | -83.824905 | 1.96% | 0.23% | motif file (matrix) |
| 6 | 0.775 | C T G A C G T A A C G T A G T C C T G A A G C T A T C G A C T G | 1e-28 | -66.534908 | 5.45% | 2.16% | motif file (matrix) |
